# Post-translational modifications in sepsis-induced organ dysfunction: mechanisms and implications

**DOI:** 10.3389/fimmu.2024.1461051

**Published:** 2024-08-21

**Authors:** Lin Song, Wei Jiang, Hua Lin, Jiangquan Yu, Ke Liu, Ruiqiang Zheng

**Affiliations:** ^1^ Northern Jiangsu People's Hospital Affiliated to Yangzhou University, Yangzhou, China; ^2^ Intensive Care Unit, Northern Jiangsu People’s Hospital, Yangzhou, China

**Keywords:** sepsis, post-translational modifications, organ dysfunction, inflammation, infection

## Abstract

As a grave and highly lethal clinical challenge, sepsis, along with its consequent multiorgan dysfunction, affects millions of people worldwide. Sepsis is a complex syndrome caused by a dysregulated host response to infection, leading to fatal organ dysfunction. An increasing body of evidence suggests that the pathogenesis of sepsis is both intricate and rapid and involves various cellular responses and signal transductions mediated by post-translational modifications (PTMs). Hence, a comprehensive understanding of the mechanisms and functions of PTMs within regulatory networks is imperative for understanding the pathological processes, diagnosis, progression, and treatment of sepsis. In this review, we provide an exhaustive and comprehensive summary of the relationship between PTMs and sepsis-induced organ dysfunction. Furthermore, we explored the potential applications of PTMs in the treatment of sepsis, offering a forward-looking perspective on the understanding of infectious diseases.

## Introduction

Sepsis, a systemic malady precipitated by the host’s dysregulated response to infection, is commonly caused by a plethora of pathogenic microorganisms and is one of the most prevalent causes of mortality within intensive care units (ICU) (1, 2). As a grave threat to life, sepsis, characterized by its complexity and mutability, has rapidly advanced, implicating multiple organ systems (3, 4). Its hallmark manifestations include endothelial dysfunction, acute lung injury, bone marrow suppression, disturbances in acid-base balance, hepatic and renal impairment, coagulopathy, and myocardial damage (5, 6). With the aging population, increasing incidence of cancer, and increasing use of invasive medical procedures, the prevalence of sepsis is on the rise, making it a prominent global public health concern (7). Despite significant advancements in our understanding of sepsis in recent years, the complex pathophysiological mechanisms involved have limited the development of effective diagnostic and therapeutic approaches to improve patient outcomes.

Post-translational modifications (PTMs) refer to a series of covalent alterations of proteins following RNA translation, representing a pivotal phase in protein biosynthesis (8). Throughout their life cycles, organisms experience PTMs, which serve to enhance the complexity of the proteome, modify the localization of associated proteins, facilitate or inhibit interactions among proteins, and activate or deactivate relevant proteins (9, 10). Increasing research has uncovered that many critical biological processes and disease occurrences are regulated not merely by the abundance of proteins but significantly by various PTMs. The most common PTMs include phosphorylation, methylation, acetylation, ubiquitination, and glycosylation, in addition to a range of novel acylations discovered in recent years, such as succinylation, crotonylation, 2-hydroxyisobutyrylation, and lactylation (11–13). In pathways related to sepsis, PTMs play a crucial role, not only by diversifying protein functions but also by acting as switches, enabling cells or organisms to respond swiftly and precisely to stress (14, 15). In recent years, the significance of PTMs in the context of sepsis-induced multi-organ dysfunction has increasingly gained attention. Therefore, understanding the characteristics and regulatory roles of these PTMs methods holds significant value for exploring diagnostic and therapeutic measures for sepsis-induced multi-organ dysfunction.

**Figure 1:**
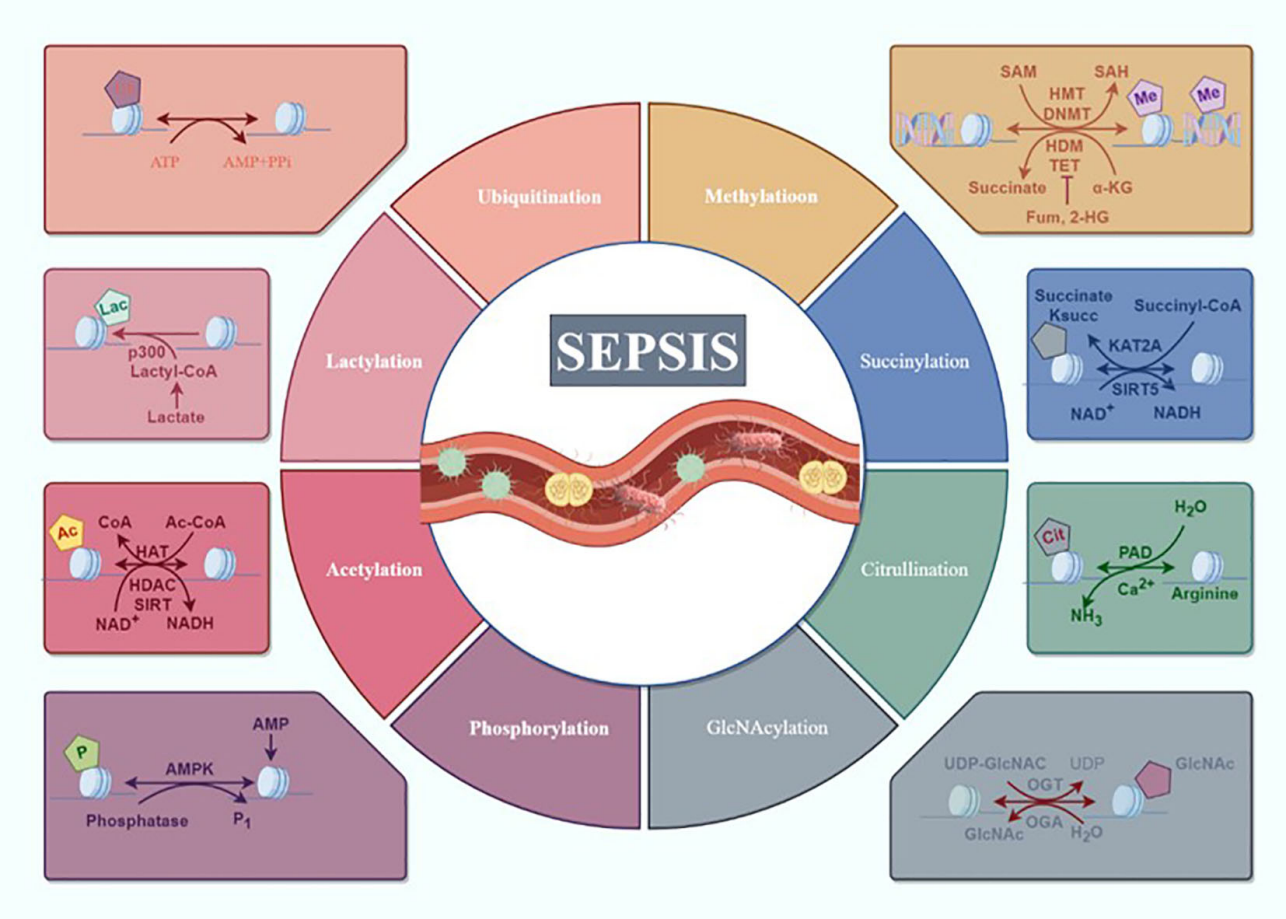
Overview of the composition and regulatory mechanisms of PTMs in sepsis. Sepsis and multiple organ dysfunction caused by the main way of protein modification after translation for protein phosphorylation, acetylation, ubiquitination, methylation and lactylation, succinylation, citrullination, GlcNAcylation may participate in the occurrence of sepsis development but has not yet been confirmed (draw by Figdraw).

In this comprehensive review, we have examined the advancements in PTMs in sepsis, summarizing recent progress in understanding how protein phosphorylation, acetylation, ubiquitination, methylation, lactylation, and so on impact the multi-organ dysfunction induced by sepsis (**Figure 1**). This paper aims to elucidate the mechanisms by which various PTMs are involved in sepsis pathogenesis and their applications in sepsis treatment, providing valuable insights for interventions in sepsis and its associated multi-organ dysfunction.

## Role of post-translational modifications in sepsis

### Regulation of protein post-translational modifications in sepsis

#### Phosphorylation

Protein phosphorylation, catalyzed by protein kinases, represents a ubiquitous type of PTMs that plays a pivotal role in the pathogenesis and progression of sepsis (16). In essence, phosphorylation refers to the process whereby proteins, under the catalysis of phosphorylases, transfer phosphate groups from adenosine triphosphate (ATP) or guanosine triphosphate (GTP) to specific amino acid residues (17). The pathogenesis of sepsis is intricate and involves multifaceted pathophysiological alterations across multiple systems and organs (18). Systemic inflammatory response syndrome triggered by infection is a hallmark of sepsis (19). While inflammation is critical for the eradication of pathogens, unbridled inflammation can precipitate the development of sepsis. Moreover, the expression of inflammatory mediators is intimately associated with protein phosphorylation. Research has revealed that recombinant homeodomain interacting protein kinase 2 (HIPK2), a serine/threonine kinase, exhibits elevated expression in inflammatory macrophages. Notably, mice deficient in *Hipk2* are more susceptible to cecal ligation and puncture (CLP)-induced sepsis, as HIPK2 can bind to and phosphorylate histone deacetylase 3 (HDAC3), inhibiting its enzymatic activity. This inhibition, in turn, suppresses the activity of P65 and curtails the activation of NF-κB, thereby mitigating infection-related septic shock (20). Krüppel-like factor 4 (KLF4) is a pivotal anti-inflammatory transcription factor that is reportedly involved in the immune response to sepsis (21). In LPS-induced RAW264.7 cells and CLP-induced septic mouse liver and lung tissues, recombinant Toll-like receptor 4 (TLR4) facilitates the phosphorylation of ERK1/2, leading to the downregulation of KLF4 and resulting in increased expression of ITGA2B (22–24). This underpins the inflammatory response in sepsis, with liver damage before and after sepsis considered to be of significant importance. Any intervention that mitigates liver damage and fosters liver function recovery can contribute to reducing mortality and morbidity rates. Furthermore, the JAK/STAT pathway plays a crucial role in the systemic inflammatory response induced by sepsis (25). Upon cytokine activation, JAKs selectively phosphorylate STAT, leading to the suppression of the JAK2/STAT3 signaling pathway. This suppression reduces the levels of TNF-α, IL-6, and IL-1β within the body during sepsis, diminishing the inflammatory response and sepsis-induced multiorgan dysfunction (26, 27). Similarly, the NLRP3 inflammasome mediates the onset of numerous inflammatory diseases, including sepsis-induced septic shock. Studies have shown that in LPS-induced septic shock, Pin1 influences the phosphorylation of p38 MAPK, activating NLRP3 inflammasome-mediated pyroptosis (28). In summary, given the heterogeneity of sepsis, its pathogenesis is complex. However, the hypothesis of target phosphorylation potentially elucidates the primary characteristic of sepsis, the inflammatory response. These findings suggest a promising role for phosphorylation in the development of well-defined therapeutic targets for sepsis.

#### Acetylation

Protein acetylation stands as a pivotal PTMs, intricately involved in regulating vital physiological processes, including but not limited to epigenetics (29). Its significance extends to chromosomal recombination, transcriptional modulation of genes, cellular metabolic regulation, dynamic control of protein stability, and the orchestrated response to microbial infections (30, 31). Aberrations in acetylations can precipitate unbridled gene expression, subsequently altering enzymatic activities in metabolism, influencing protein-protein interactions, and compromising protein stability (32). Such anomalies instigate aberrant cell proliferation, thereby triggering the onset of developmental and proliferative disorders. Dysregulation of protein acetylation pathways becomes a player in sepsis pathogenesis, influencing gene expression patterns, modulating crucial signaling pathways, and ultimately contributing to the dysfunctions observed in various organs during this critical condition. In recent years, an increasing body of experimental evidence has underscored the indispensable role of deacetylase enzymes known as sirtuins in regulating inflammatory responses and the progression of sepsis (33, 34).SIRT1 plays diametrically opposing roles during the high and low inflammatory response phases of sepsis (35). Its expression is typically subdued in the initial stages of sepsis, yet the heightened glycolysis triggered by the intense inflammatory response can lead to an accumulation of niacinamide adenine dinucleotide (NAD^+^), thereby promoting the upregulation of SIRT1 expression. Through deacetylating inflammatory pathways involving factors like NF-κB/p65 and high mobility group box 1 (HMGB1), SIRT1 exerts its anti-inflammatory effects (36). In a mouse model of sepsis, through deacetylation of NF-κB, SIRT1 renders it inactive, thereby diminishing pro-inflammatory response levels. However, the absence of SIRT1 promotes Akt acetylation, consequently enhancing the production of inflammatory factors in murine macrophages, potentially exacerbating the progression of sepsis in mice (37, 38). SIRT2, in addition to its deacetylation role on NF-κB/p65 akin to SIRT1 in acute inflammation, holds particular significance in regulating microvascular inflammation and promoting low inflammation in septic obese mice (39). SIRT5 competes with SIRT2 in its interaction with NF-κB/p65, as SIRT5 can interact with NF-κB/p65 in a deacetylase activity-independent way, thereby impeding SIRT2’s deacetylation of p65 and fostering the activation of NF-κB and its downstream signaling molecules (40). In a rodent model of sepsis-induced acute kidney injury, a reduction in the activities of SIRT1 and SIRT3 led to heightened acetylation levels of SOD2, accompanied by oxidative stress and mitochondrial impairment (41). Acting as an upstream deacetylase, SIRT3 regulates the acetylation level of pyruvate dehydrogenase E1 component subunit alpha (PDHA1) in renal tubular cells, participating in the occurrence and development of sepsis-associated acute kidney injury (SAKI). Downregulation of SIRT3 expression in renal tissues and HK-2 cells after sepsis results in an elevation of PDHA1 acetylation levels coupled with diminished PDHA1 activity (42). Research has also revealed that SIRT6, SIRT1, and NF-κB collaborate in orchestrating the metabolic reprogramming of energy during the pathophysiological processes of sepsis (43). Thus the function of different Sirtuins in sepsis suggests that targeting Sirtuin family members at different stages of sepsis can help to precisely control the progression of sepsis. Moreover, in a rat model of sepsis-associated encephalopathy (SAE), cognitive function declines, accompanied by increased expression and acetylation levels of cyclophilin D (CypD) and HMGB1, as well as elevated expression of inflammatory factors such as IL-6 and TNF-α (44, 45). HMGB1, a crucial product of the inflammatory response, can trigger neuroinflammation by transporting and activating macrophages derived from the bone marrow into the brain, leading to postoperative cognitive dysfunction (46, 47). The HMGB1 inhibitor sodium butyrate can suppress the expression of HMGB1, IL-6, and acetylated HMGB1; elevate the levels of brain-derived neurotrophic factor (BDNF); and significantly ameliorate cognitive dysfunction induced by SAE (48). Protein acetylation has emerged as a critical facet of sepsis and is intricately interwoven with the complex tapestry of molecular responses. These PTMs assume a central role in orchestrating cellular reactions during the tumultuous cascade of events characterizing sepsis. Its involvement extends beyond the traditional realms, reaching into the intricate web of immune responses, inflammatory signaling, and the delicate equilibrium of cellular homeostasis.

### Ubiquitination and SUMOylation

Ubiquitination refers to the attachment of small ubiquitin proteins to target proteins, regulating protein stability, activity, and interactions through this covalent modification (49, 50). Research has revealed a significant increase in the ubiquitination levels of proteins, particularly immune cells and inflammation-related proteins, in sepsis (51, 52). Ubiquitination plays a pivotal role in regulating inflammation by modulating the activity of inflammatory signaling pathways (53–55). In a mouse model of LPS-induced sepsis-induced lung injury, reducing TRIM27 was found to alleviate sepsis-induced inflammation, oxidative stress, and cell apoptosis by inhibiting PPARγ ubiquitination and decreasing NADPH oxidase 4 (NOX4) expression (56). Similarly, this manifestation of sepsis can also lead to a significant decline in cardiac function. Research has revealed increased expression of CCAAT/enhancer-binding protein beta (CEBPB) in LPS-induced mouse myocardial tissue. This heightened expression triggers macrophage-mediated inflammation and the onset of systemic inflammatory and microvascular injury (SIMI). However, the ubiquitin modification of CEBPB by the constitutive photomorphogenesis protein 1 homolog (COP1) results in CEBPB degradation and the inhibition of macrophage inflammatory responses (57). The ubiquitin modification of CEBPB acts as a safeguard, protecting the heart from the ravages of SIMI (58). On the other hand, ubiquitination plays a widespread role in regulating appropriate immune responses (59). Recent studies have revealed the crucial role of hypoxia-inducible factor-1α (HIF-1α) in adaptive and cell-protective responses, in cell survival, proliferation, apoptosis, inflammation, and angiogenesis (60, 61). In the early stages of sepsis, activating transcription factor 4 (ATF4) regulates macrophage pro-inflammatory response activation through direct targeting of HK2 or interaction with HIF-1α to maintain its stability. Inducing ATF4 expression can induce immune activation in tolerant macrophages, providing new insights for immune reconstitution therapy in sepsis (62). Endothelial cell dysfunction and immune dysregulation are considered pivotal pathogenic features of sepsis (63). Research has revealed that TRIpartite Motif-containing (TRIM), as a subfamily of E3 ubiquitin ligases, participates in cell proliferation and differentiation, as well as in maintaining endothelial function. TRIM8 regulates the activation of TNF-α and IL-1β through mediating the multiubiquitination of TAK1 with K63 linkage, thus modulating the NF-κB signaling pathway (64). TRIM2 alleviates sepsis-induced endothelial cell damage by inhibiting the NF-κB pathway and the release of inflammatory factors (65). TRIM47 acts as a stimulator of TNF-α-induced endothelial cell activation and, as a potential E3 ubiquitin ligase, interacts with TNF receptor associated factor 2 (TRAF2), mediating K63-linked ubiquitination to activate the NF-κB signaling pathway (66).In summary, protein ubiquitination plays a crucial role in sepsis and the resulting multi-organ dysfunction. A deeper exploration of the molecular mechanisms of ubiquitination will offer new perspectives for understanding the pathological processes of sepsis, providing more precise targets for future therapeutic strategies. With ongoing scientific advancements, we anticipate uncovering the intricate interplay between protein ubiquitination modification and sepsis.

In addition to ubiquitination, there are reactions similar to ubiquitination modifications, such as small ubiquitin-like modifier (SUMOylation). It regulates cell signaling, gene transcription, cell proliferation, and apoptosis by covalently attaching SUMO proteins to specific proteins, playing an important role in regulating inflammation and immune responses in sepsis (67). Ubiquitin-conjugating enzyme 9 (UBC9) is the sole SUMO ligase for SUMOylation, and changes in UBC9 expression directly reflect SUMOylation capacity (68). Studies have shown that LPS-induced UBC9 gene knockout in mice increases mortality in a sepsis model, and *in vitro* cell experiments have revealed that UBC9 deficiency accelerates dendritic cell (DC) maturation and enhances inflammatory responses. Similarly, UBC9^ΔDC^ mice in the cecal ligation and puncture (CLP) sepsis model exhibit higher mortality rates than WT mice in the CLP sepsis model, with significantly elevated levels of IL-18 and IL-1β in plasma. While SUMOylation has no effect on DC, its absence may increase sepsis mouse mortality by regulating DC inflammatory cytokine release and abnormal T cell activation, confirming that SUMOylation may be a protective factor in sepsis (69). Macrophage dysfunction is considered a significant factor affecting immune homeostasis and the inflammatory process in sepsis, and the role and related mechanisms of SUMOylation in macrophage inflammation during sepsis have been confirmed (70). SUMO specific peptidase 1 (SENP 1) is involved in the inflammatory response processes of various cells, with notably increased expression of SUMO specific peptidase 1 (SENP1)in RAW 264.7 cells induced by LPS. SENP1 promotes Sp3 expression through deSUMOylation and interaction with NF-κB, enhancing LPS-induced macrophage inflammation (71). Recent studies have reported that ginkgolic acid (GA) increases inflammation and apoptosis in sepsis mouse macrophages, leading to organ damage, possibly by inhibiting the SUMOylation process and increasing NF-κB/p65 phosphorylation and nuclear translocation. However, further validation using SUMOylation activators is needed to investigate the interaction of SUMOylation with macrophages in sepsis (72). Research on SUMOylation has been limited due to a lack of specific inhibitors, until the appearance of the first selective SUMO inhibitor, TAK981, which has provided new insights into the clinical significance of SUMOylation given its connection to cancer and sepsis (73). Youssef et al. have focused on the impact of TAK981 on endotoxin immune responses, and results have shown that recurrent TAK981 enhances early TNF-α production. In the spleen, sepsis induces a significant time and substrate specificity of SUMO1 and SUMO2/3, both of which are inhibited by TAK981 (74). The therapeutic effects of TAK981 in cancer treatment have entered clinical trials, making new immunotherapies and anti-tumor treatments possible, and providing new clinical opportunities for infectious diseases and sepsis treatment (75). In conclusion, further research on the mechanisms of SUMOylation in inflammation and sepsis will help uncover the pathogenesis of diseases and provide an important theoretical basis for developing new treatment strategies.

Although the mechanisms of ubiquitination and SUMOylation are different, in some cases, SUMOylation can induce proteins to be degraded through the ubiquitination pathway. E3 ubiquitin ligases containing SUMO-interacting motifs can recognize and bind to proteins modified by SUMOylation, thereby inducing unmodified lysine residues to be degraded through the ubiquitination pathway (76). Proteomic experiments have also confirmed that the SUMOylation status can be altered by ubiquitination (77). However, there have been no reports of relevant studies in sepsis, so further research is necessary to elucidate the balance of ubiquitination and SUMOylation in sepsis, especially in infection-inflammatory events. Additionally, providing a more detailed description of the key components of ubiquitination and SUMOylation will help identify new molecular targets for early diagnosis and treatment of sepsis.

### Lactylation

As a byproduct of glycolysis, lactate has long been perceived as a metabolic byproduct (78, 79). However, with scientific advancements, researchers have embarked on the reassessment of lactic acid, unveiling its crucial roles in serving as both fuel and signaling molecules (80, 81), promoting vascular genesis (82, 83), and inhibiting immune cells (84, 85). However, the molecular mechanisms underlying the regulation of these biological functions by lactate remain elusive. Recently, lactylation modification has emerged as a novel protein alteration that facilitates gene regulation through the covalent coupling of lactyl groups with lysine residues in proteins (12, 86). As a PTM of both proteins and metabolic byproducts, lactylation is often intricately linked with energy metabolism and cellular redox status (87). It plays a multifaceted role in the pathogenesis of sepsis, contributing to a complex interplay in its mechanistic landscape. In clinical practice, elevated lactate levels are intricately linked to both the incidence and prognosis of SAKI (88). A surge in lactate serves as an independent risk factor for patients suffering from SAKI (89). Research has revealed that the escalation of endogenous lactate and exogenous lactate supplementation intensifies the levels of nonhistone Fis1 in renal tubular epithelial cells, exacerbating SAKI. Conversely, reducing lactate levels and fission 1 protein (Fis1) lactylation mitigates SAKI, revealing a novel mechanism linking lactate and organ damage in sepsis (42). This study underscores the significance of therapeutic strategies aimed at lowering blood lactate levels in septic patients. While research on histone lactylation is more prevalent, beyond the investigation of nonhistone Fis1 lactylation, macrophage-derived nonhistone HMGB1 lactylation in sepsis has also been reported. Clinical evidence indicates a significant increase in circulating HMGB1 levels, which is positively correlated with the severity of sepsis and mortality rate (80, 90, 91). Studies have revealed that lactate promotes the lactylation of macrophage HMGB1 in polymicrobial sepsis and that reducing lactate production or inhibiting GPR81-mediated signaling can decrease extracellular HMGB1 levels, enhancing the survival outcomes of polymicrobial sepsis patients (92). Importantly, the elevation of lactate levels in septic patients is often accompanied by the release of inflammatory factors (93). This inflammatory state is intricately linked to increased lactylation, resulting in the formation of a complex regulatory network that may play a pivotal role in the transmission and maintenance of inflammatory responses. Additionally, research has revealed a significant increase in histone H3K18 lactylation in the peripheral blood mononuclear cells of septic shock patients. This increase correlated positively with the APACHE II score, SOFA score on day 1, duration of ICU stay, duration of mechanical ventilation, serum lactate level, and production of inflammatory cytokines. H3K18 lactylation may serve as a reflection of the severity of critical illnesses and the presence of infection (94). While the exploration of lactylation has only commenced within the past three years, it has already demonstrated significant potential. A profound understanding of the intricate interplay between lactylation, inflammation, multiorgan dysfunction, and immune modulation is emerging. This understanding holds promise for revealing novel perspectives for the diagnosis, treatment, and prognosis of sepsis. Subsequent research endeavors are poised to expand this field, providing further insights into the intricate relationship between lactylation modification and the pathogenesis of sepsis. The entire research on lactylation is still in its infancy, with several unresolved issues. For instance: 1. Whether lactylation is a natural consequence of high lactate accumulation in sepsis, caused by tissue hypoperfusion, abnormal cell metabolism, or impaired liver and kidney function, or is it a meticulously controlled mode regulated by time and space? 2. Clinical translation of lactylation in sepsis poses significant challenges, as individual differences among patients may affect the level and efficacy of lactylation. Sepsis is a dynamic process, with potentially varying patterns of lactylation at different stages, adding complexity to its application in clinical practice. 3. Identifying lactylation sites across multiple systems and establishing a comprehensive multi-species lactylation map library can greatly advance research on lactylation. This will ultimately enhance our understanding of lactylation function and pave the way for further exploration in this field. Future studies will continue to expand in this area, providing more insights into the intricate relationship between lactylation and sepsis.

### Methylation

Methylation is a pivotal mode of protein and nucleic acid modification, with the ability to orchestrate the expression and silencing of genes (95). Methylation is intimately intertwined with a spectrum of ailments, such as cancer (96), aging (97), and neurodegenerative disorders (98), and has emerged as a focal point within the realm of epigenetics. The most prevalent forms of methylation are DNA methylation and histone methylation. Notably, histone methylation, a significant PTM, primarily occurs through the catalytic process of histone methyltransferases (HMTs), which act upon the side chains of lysine and arginine residues (99). This process is commonly associated with the modulation of transcriptional activation or inhibition (100, 101). In sepsis, the release of inflammatory factors and activation of immune cells may induce changes in intracellular signaling pathways, influencing the methylation status of proteins (102). A study involving human monocytic cell lines revealed that LPS stimulation could induce methylation of the tumor necrosis factor (TNF) promoter. Simultaneously, this leads to the departure of nucleosomes from the NF-κB binding site of the TNF promoter region. This epigenetic modification promoted the association of NF-κB with the TNF promoter, resulting in the upregulation of TNF transcription (103). Another investigation revealed a close correlation between the DNA methylation profile of monocytes in septic patients and interleukin levels in circulating leukocytes. This association was mediated through Toll-like receptors and downstream inflammatory pathways, providing further evidence of the intricate relationship between DNA methylation and inflammatory responses (104, 105). These findings collectively underscore the relevance of DNA methylation in the context of inflammatory reactions.

Furthermore, protein methylations may play a regulatory role in modulating the functionality of immune cells by controlling gene expression. During the progression of sepsis, the activation of immune cells and the release of inflammatory factors exert a widespread and sustained impact on the organism (106). Methylations may act as modulators in this process, influencing the transcriptional levels of genes and regulating the activity of immune cells (103). Prolonged exposure to toxins can lead to a sustained increase in TNF-α, resulting in the depletion of the transcription factor megakaryocytic leukemia 1 (MKL1). MKL1 is essential for H3K4 dimethylation and trimethylation in the NF-κB promoter region induced by LPS. Depletion of MKL1 directly leads to a reduction in NF-κB expression levels, resulting in endotoxin tolerance (107). Continuous endotoxin stimulation also induces the recruitment of histone methyltransferase G9a to the TNF promoter region, causing H3K9 dimethylation and recruiting DNA (cytosine-5)-methyltransferase 1 (DNMT-1), subsequently leading to the remethylation of the TNF promoter. After remethylation of the TNF promoter, the promoter becomes insensitive to endotoxin stimulation, and even exposure to endotoxin cannot induce demethylation activation, resulting in the development of endotoxin tolerance in the organism (108). In summary, research related to the epigenetics of sepsis primarily consists of animal experiments or *in vitro* studies, with limited data from *in vivo* experiments. The exploration of the complete epigenome of sepsis patients has begun, but further in-depth research is needed to gain a comprehensive understanding of the regulatory role of methylations in sepsis. This deeper understanding holds the potential to provide a novel theoretical foundation for the development of more effective therapeutic strategies in the future.

### Citrullination,succinylation,GlcNAcylation

Beyond the aforementioned protein modifications, which have been extensively studied, there exist other modifications of equal importance that remain underexplored in the context of sepsis.

Citrullination constitutes a physiological post-translational modification whereby, catalyzed by Ca^2+^-dependent peptidylarginine deiminase (PAD), arginine is converted into citrulline (109, 110). This process is critical for modulating chromatin remodeling and the extracellular trap formation of immune cells. Citrullination of histones, especially histone H3, has been exposed as a cog in the wheel of neutrophil response to infection, embodying an array of inflammatory signals (111, 112). The enzyme peptidylarginine deiminase 4 (PAD4) activity is an essential component of histone citrullination and chromatin decondensation, crucial steps required for neutrophil extracellular trap formation (NETosis) (113, 114). The levels of citrullinated H3 (CitH3) are dependent on PAD4 activity, as evidenced by reduced levels of pro-inflammatory factors and neutrophil infiltration in the lungs of PADI4^-/-^ mice (115). Interestingly, studies have shown that vitamin C can exert a protective effect against NETosis and sepsis formation mediated by PAD4 inhibition of CitH3 (116). Critically, specific inhibitors of PAD4 are in development and are widely used in the study of NETosis in sepsis, which is important for gaining insight into the role of NETosis formation in critically ill patients (117). Actually, Levels of CitH3 within the circulation have been also verified as early diagnostic markers for sepsis, with burgeoning studies identifying a correlation between systemic dysfunction in sepsis and elevated serum levels of CitH3, reflecting the gravity of septic conditions (118–120). In a murine model of sepsis, heightened levels of CitH3 are associated with sepsis-induced acute respiratory distress syndrome and lung dysfunction. CitH3 is also known to activate the caspase-1-dependent inflammasomes in myeloid-derived macrophages and dendritic cells, leading to acute lung injury (ALI). Conversely, studies have corroborated that therapeutic administration of monoclonal antibodies against CitH3 substantially ameliorates survival rates in septic mice while mitigating ALI, potentially due to inhibition of CitH3-activated caspase-1-dependent inflammasome pathways. Targeting the CitH3-caspase-1 axis may represent a promising therapeutic avenue for septic shock and sepsis-induced ALI (121).

Succinylation constitutes a process whereby succinyl groups are covalently bonded to lysine residues via enzymatic or non-enzymatic means, affecting protein structure and function, and thereby regulating signaling pathways and cellular metabolism intrinsic to life (122, 123). The role of succinylation in sepsis and related ailments has been the subject of scant research. Studies in a mouse model of burn-induced sepsis have revealed that glutamine augments the activity of pyruvate dehydrogenase (PDH) in macrophages; mechanistically, glutamine attenuates SIRT5-dependent desuccinylation of PDHA1, thus reinstating PDH activity, bolstering M2 polarization in macrophages, and ameliorating burn-induced sepsis in mice. This research not only affirms the fundamental role of glutamine in supporting M2 polarization in macrophages and treating burns and their complications but also offers novel insights into the function of succinylation in the context of sepsis (124).

GlcNAcylation represents an essential mode of post-translational modification. Through this adjustment, sugar moieties are covalently bonded to specific residues on proteins by enzymatic catalysis—forming glycosidic linkages (125, 126). Engaged in a myriad of biological processes, glycosylation modulates protein folding, cell signaling, differentiation, and immune responses. In mammals, protein glycosylation manifests primarily in two forms: N-GlcNAcylation and O-GlcNAcylation, the latter of which, specifically the O-GlcNAcylation signaling, is sensitive to diverse forms of stress-induced sepsis and shock under various pathological conditions (127, 128). In the early stages of sepsis, O-GlcNAc signaling enhancement ameliorates outcomes in septic shock by preserving cardiovascular function, a phenomenon tied to the recuperation of sarcoplasmic/endoplasmic reticulum calcium ATPase2a (SERCA2a) levels (129). Similarly, O-GlcNAc stimulation not only offers protection to adult rats with sepsis but also improves prognoses in their juvenile counterparts (130, 131). O-GlcNAc transferase (OGT), the pivotal enzyme for O-GlcNAcylation, plays a significant role; its deficiency leads to augmented innate immune activation and exacerbated septic inflammation. A recent study unveiled that OGT-mediated O-GlcNAcylation of serine-threonine kinase receptor-interacting protein kinase 3 (RIPK3) hinders ectopic RIPK3-RIPK1 and homotypic RIPK3-RIPK3 interactions, thereby curtailing downstream innate immune and necroptotic signaling (132). This research underscores the importance of O-GlcNAcylation in septic inflammation and paves the way for potential therapeutic interventions against sepsis.

## Effect of RNA modifications in sepsis

Although both RNA modification and post-translational protein modification involve the process of modifying biomolecules, they occur separately in RNA and protein molecules and are involved in distinct biological processes (133). RNA modification refers to the chemical alteration of nucleotides in RNA molecules, a process that can regulate the stability, readability, and functionality of RNA (134). Common modifications include N6-methyladenosine (m6A) modification, 5-methylcytosine (m5C) modification, and ADP-ribose nucleic acid (ADAR) modification, among others (135). In sepsis, the levels of m6A modifications may change, influencing the expression of genes associated with immunity and inflammation. This modification affects the development of sepsis by regulating the production of inflammatory factors, activating cell signaling pathways, and modulating the functionality of immune cells (**Table 1**) (140, 141).

**Table 1:** Role of N6 methylation in sepsis.

PTMs	Disease	Main Object	Mechanism	Reference
N6-methyladenylate	Sepsis-induced myocardial injury	Mice; Macrophages	Exposure to LPS upregulates the lysine acetyltransferase, KAT2B, to promote METTL14 protein stability through acetylation at K398, leading to the increased m^6^A methylation of Spi2a in macrophages. m^6^A-methylated Spi2a directly binds to IKKβ to impair IKK complex formation and inactivate the NF-κB pathway.	(136)
	Sepsis-induced lung injury	Mice; Macrophages	mitophagy induced the demethylation of the miR-138-5p promoter, which may subsequently inhibit NLRP3 inflammasome, AM pyroptosis and inflammation in sepsis-induced lung injury.	(137)
	Sepsis-induced myocardial injury	Rat; cardiomyocyte (H9C2) cells	The m^6^A modified SLC7A11 mRNA was recognized by YTHDF2, which promoted the decay of SLC7A11 mRNA, consequently up-regulating ferroptosis in sepsis-induced myocardial injury.	(138)
	Sepsis-induced myocardial injury	Rat; cardiomyocyte (H9C2) cells	HDAC4 had remarkable m^6^A modification sites on its 3'-UTR genome, acting as the downstream target of METTL3. Besides, m^6^A reader IGF2BP1 recognized the m^6^A modification sites on HDAC4 mRNA and enhanced its RNA stability.	(139)

## N6-methyladenylate

Methylation, in addition to protein methylation, extends to m6A methylation—a modification primarily occurring on adenosine bases within RNA molecules. It plays a pivotal role in regulating the stability, transport, translation, and degradation of RNA (142, 143). In recent years, researchers have shown a keen interest in the role of m6A modification in various physiological and pathological processes (144). Within the context of sepsis, alterations in the RNA levels of m6A modifications may occur, influencing the expression of genes associated with inflammation. In sepsis, alterations in the m6A modification of RNA may occur, influencing the expression of inflammation-related genes (145). Research indicates that m6A methylation can modulate the decay and translation efficiency of Spi2a (136). Loss of RNA methylation in macrophages exacerbates cytokine storms and sepsis-related myocardial dysfunction. m6A modification also plays a role in regulating the function of immune cells. Clinical studies have identified genes such as WTAP and methyltransferase-like protein 16 (METTL16), which, through the modulation of m6A methylation, facilitate immune cell infiltration, accelerating the onset and progression of sepsis in both healthy individuals and late-stage sepsis patients (146). These genes provide potential drug targets for the early detection, diagnosis, and treatment of sepsis. Recent research has demonstrated a direct role for m6A methylation in LPS-induced pulmonary inflammatory lesions. Elevated m6A methylation levels of circN4BP1 were observed in mice with sepsis-induced respiratory distress syndrome (ARDS). CircN4BP1, by binding to miR-138-5p, influences macrophage differentiation, thereby regulating the expression of zeste homolog 2 (EZH2) both *in vivo* and *in vitro (* 137). Moreover, the mRNA levels of the m6A-related enzymes METTL3, FTO, and YTHDF2 also significantly increased. Knocking out METTL3 markedly reduced circN4BP1 expression, regulating downstream EZH2 expression, further influencing macrophage polarization, and ameliorating the inflammatory response induced by sepsis-induced ARDS (138). Despite our initial understanding of the role of m6A methylation in sepsis, many unknown areas remain that require further exploration. In the same way, METTL3 can regulate sepsis-induced myocardial injury through an IGF2BP1/HDAC4-dependent mechanism, and knockdown of METTL3 greatly inhibits myocardial cell damage (139). Research may focus on specific targets of m6A methylation, elucidating the underlying mechanisms, and exploring potential therapeutic strategies targeting this modification in the future.

In the context of sepsis, investigations into RNA modifications remain in their nascent stage. Despite the discovery of some modifications associated with inflammation and immune responses, a more profound understanding of their precise roles in the pathophysiology of sepsis is imperative. The challenges in this realm encompass both technical intricacies and the necessity for a comprehensive comprehension of modification effects. With the continual evolution of technology, we anticipate further insights into the potential roles of RNA modifications in sepsis, thereby offering insight into disease mechanisms and therapeutic strategies.

## Interaction of different PTMs in sepsis

In conclusion, the ubiquity and dynamics of PTMs imply their involvement in various aspects of sepsis pathogenesis, where different types of PTMs interact and coordinate complex pathophysiological functions (147). PTMs at different sites of the same protein may have different effects on diseases. Various PTMs processes do not exist in isolation; in many cellular activities, proteins requiring various post-translational modifications act in concert (148). Existing research has confirmed a close relationship between lactylation and acetylation. Circulating HMGB1, as an important damage-associated molecular pattern (DAMP) molecule, plays a crucial role in the progression and late-stage mortality of sepsis (149, 150). Studies have found that HMGB1 can undergo lactylation and acetylation, participating in sepsis development (92). Macrophages uptake extracellular lactate via Monocarboxylate Transporters (MCTs), promoting lactylation of HMGB1 through a p300/CBP-dependent mechanism. Lactate inhibits HMGB1 acetylation through Hippo/Yap-mediated deacetylase SIRT1 suppression and stimulates HMGB1 acetylation through β-arrestin2-mediated acetyltransferase p300/CBP recruitment to the nucleus via G protein-coupled receptor 81 (GPR81). Lactylated/acetylated HMGB1 is released from macrophages by exosomes, increasing endothelial cell permeability. Decreased intracellular lactate production or inhibition of the GPR81-mediated signaling pathway reduces extracellular exosomal HMGB1 levels (92). Histones can be co-modified by methylation and acetylation. The major acetylation and methylation sites on histones H3 and H4 are conserved lysine residues at the C-terminus. Histone acetylation modification occurs throughout the cell cycle, while methylation modification often occurs during the G2 phase and chromatin assembly process. Studies show that in LPS-stimulated endothelial cells, the acetylation levels of active histone aceH3K9 and aceH3K18 in the VE-cadherin promoter decrease, while the acetylation levels increase in cells treated with DNA methyl transferase inhibitor 5-Aza 2-deoxycytidine(Aza) and histone deacetylase inhibitor trichostatin (TSA), and the methylation levels of me2H3K9 decrease after treatment with Aza and TSA, indicating that the protective effect of Aza and TSA combined therapy on the endothelial barrier is the result of histone acetylation and methylation modifications at the VE-cadherin promoter level (151). These experiments demonstrate the widespread possibility of interactions between various modifications, and the potential synergistic or competitive relationships between multiple modifications in regulating protein function in sepsis are worth further exploration. In sepsis-induced multi-organ dysfunction, it is necessary to continue exploring whether the occurrence of PTMs in different organs and the ratios between different PTMs affect the onset and progression of the disease.

## Summary and perspectives

**Figure 2:**
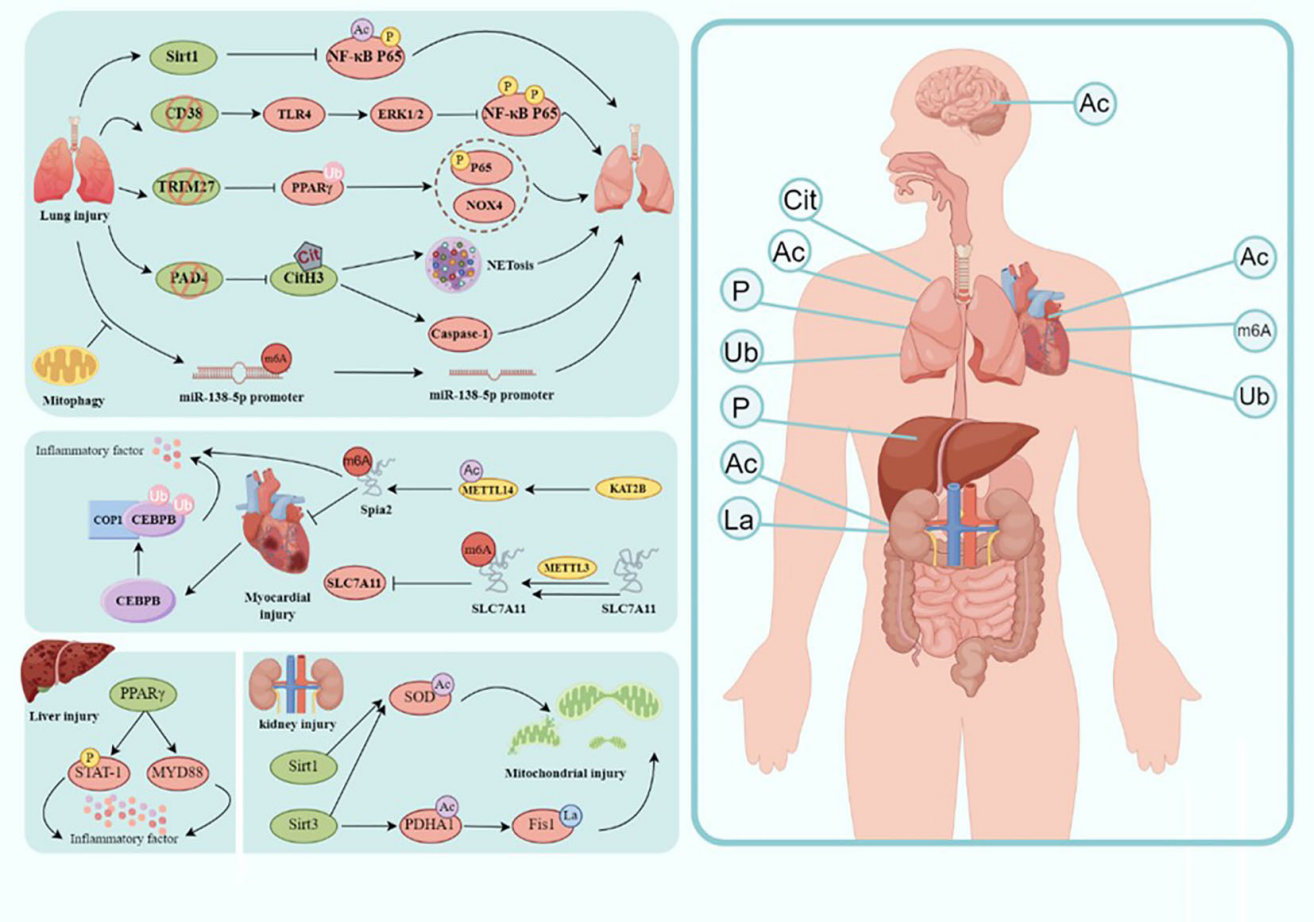
Modification in the molecular mechanism of sepsis-induced organ injury. Involvement of sepsis common organs including the brain, heart, lungs, liver, kidney, intestine, etc., and different modification happens in different organs. Ac, acetylation; Cit, citrullination; P, phosphorylation; La, lactylation; Ub, ubiquitination(draw by Figdraw).

Sepsis is a systemic inflammatory response syndrome caused by an infection that often leads to multiple organ dysfunction and is a life-threatening condition (152). Due to the elusive nature of its specific pathogenesis, the treatment of sepsis has remained a focal point in the field of medicine. Currently, the therapeutic approach for sepsis primarily emphasizes comprehensive interventions on various fronts, including antibiotic therapy, fluid resuscitation, hemodynamic support, immune modulation, and organ support (153, 154). However, owing to the complexity of the pathophysiology of sepsis, the limitations of conventional treatment strategies are gradually becoming evident. Anti-infective therapy is the foremost step in sepsis treatment, with the early use of broad-spectrum antibiotics aiding in swiftly controlling the source of infection and halting its further spread (155). Nevertheless, due to the misuse of antibiotics and the escalating issue of resistance, the effectiveness of anti-infective treatment is progressively constrained. Furthermore, traditional anti-inflammatory treatments, such as glucocorticoids, can partially suppress the inflammatory response; however, their broad-spectrum immunosuppressive effects have a range of side effects (156). Therefore, the imperative to explore new treatment strategies has emerged, and PTMs, as emerging therapeutic targets, have garnered widespread attention. PTMs are crucial mechanisms regulating the function of RNA molecules and proteins, displaying remarkable diversity and close associations with various diseases. Decades of research have confirmed the vital roles played by phosphorylation, acetylation, methylation, ubiquitination, and others in numerous biological processes. As described in this article, an increasing number of researchers are exploring the potential significance of PTMs in sepsis and sepsis-induced multiple-organ dysfunction (**Figure 2**). This opens up valuable avenues for innovative research on the pathogenesis and targeted treatment of sepsis-induced multiple organ dysfunction.

Currently, some studies have delved into drugs targeting PTMs induced by sepsis. Historically, the ability to regulate histone and non-histone acetylation with HDAC inhibitors (HDACi) has been considered an effective anti-inflammatory mechanism (157). Treatment with pan-HDACi and certain subtype-selective HDACi can confer a survival advantage and reduce the expression of pro-inflammatory mediators (158). The researchers explored the potential renal protective effects of the selective class IIa HDACi TMP195 in an LPS-induced AKI mouse model (159). In experiments with bone marrow-derived macrophages (BMDMs), the HDACi SAHA reduced oxidative stress and extracellular ATP levels, ultimately dampening inflammasome activation, shedding light on the therapeutic mechanism of SAHA in sepsis treatment (160). The SUMO inhibitor TAK981 is undergoing clinical trials for cancer treatment, providing a new therapeutic strategy for sepsis (74). Another treatment approach is gene therapy, which involves repairing or regulating specific PTMs-related genes through gene editing techniques. Gene therapy is considered one of the most promising new treatments. CRISPR/Cas9 has created cell and animal models targeting many genes, opening doors to a new class of therapies. Studies have shown that using CRISPR/Cas9 gene knockout to simulate the ITCH-UBCH7 inhibitory state, hereditary UbcH7 deficiency affects ITCH phosphorylation in regulating RIPK2 ubiquitination, which disrupts the binding of E3 ubiquitin ligase to E2 binding enzyme, prolonging inflammatory signal transduction (161). While CRISPR/Cas9 technology may be the simplest and most effective way to conduct sepsis-related research, further development is needed due to the limitations in targeting genes and the instability of the technology for CRISPR/Cas9 in sepsis therapy (162). Research on sepsis is ongoing and has made some progress. While effective drugs for treating sepsis have yet to be found, promising clinical trials are underway for IκBa kinase inhibitors (SAR113945) in treating inflammatory diseases, suggesting that effective IκBa kinase inhibitors may become valuable treatments for sepsis-related inflammatory responses in the near future (163). With a deepening understanding of gene regulation in sepsis, we have reason to believe that more clinical studies will be implemented to explore effective approaches for sepsis treatment. Research on the role of PTMs in sepsis has made some progress, yet the specific molecular mechanisms involved await further clarification. Researchers can analyze the PTM activities of key proteins in regulatory pathways, their relationships with sepsis, and the underlying mechanisms involved. Based on this foundation, proactive intervention measures targeting sepsis can ultimately ameliorate its symptoms.

Although PTMs present immense potential as novel therapeutic targets for sepsis, they currently encounter a series of challenges. At present, research on the PTMs underlying organ dysfunction induced by sepsis primarily relies on animal models and *in vitro* experiments (164, 165). However, these models may not fully replicate the complex pathophysiological processes of human sepsis, thus the reliability and generalizability of existing research results are questionable (166–168). Therefore, the development of animal models more suitable for simulating human sepsis, combined with *in vitro* organ models, is essential to more accurately investigate the role of PTMs in organ failure. While some PTMs changes related to sepsis have been identified, understanding of how these changes affect organ function and their interactions in different organs remains limited (169). Through in-depth investigation of the signaling pathways and molecular mechanisms regulating post-translational modifications, uncovering their specific roles in sepsis-induced organ dysfunction can provide a clearer theoretical basis for the development of relevant drugs and treatment strategies.

The research of PTMs is a rapidly evolving field. With the continuous advancement of high-throughput omics technologies and highly sensitive mass spectrometry techniques, novel PTMs are being successively reported, providing important tools for a deeper understanding of cellular functions and disease mechanisms (170–172). However, unraveling the intricate world of protein modifications within the human body still requires relentless efforts. Now, the limitations of PTMs technologies mainly manifest in technical challenges (173), including 1. insufficient resolution and sensitivity, some detection methods may lack the necessary resolution and sensitivity to accurately detect and identify low-abundance protein post-translational modifications. 2. specificity issues, certain detection methods may have specificity problems, leading to false positives or negatives in post-translational modifications. 3. complex sample handling, complex samples may contain various types of PTMs, requiring highly specific detection methods to distinguish different types of modifications. Additionally, there are potential biases such as: 1. data interpretation bias, researchers’ subjective awareness, and preconceptions may affect the interpretation and analysis of PTMs data, leading to erroneous conclusions. Furthermore, integrating data from genomics, transcriptomics, proteomics, and metabolomics can help comprehensively understand the role of PTMs in sepsis-induced organ dysfunction. 2. sample preparation bias, improper sample handling, and preparation may lead to loss or increase of PTMs, affecting the accuracy of research results. Sepsis is a complex disease involving multiple cells and tissues, thus requiring various types of samples such as blood, tissues, and cells. However, sample collection and processing may be subject to limitations such as insufficient sample quality and quantity, as well as errors during collection that could affect the reliability of research results. 3. publication bias, publication bias may impact the reporting of research results, with researchers more likely to publish positive results, while negative results are less likely to be published, potentially leading to a misunderstanding of the overall situation in the research field. In short, continuous development and improvement of detection technologies are needed to overcome these limitations, in order to more comprehensively understand the functions and mechanisms of proteins. This is crucial for an in-depth study of how these PTMs reveal the mechanisms of life, screen clinical biomarkers, identify drug targets, and more.

In summary, the future of sepsis treatment heralds an era of comprehensive therapeutic strategies, where the combined application of traditional treatments and emerging PTM interventions holds promise for providing patients with more comprehensive and personalized therapeutic approaches. Through continuous and in-depth research, we aspire to pave the way for new avenues in sepsis treatment, bringing about improved clinical outcomes for patients.
